# Effect of Sodium Disilicate and Metasilicate on the Microstructure and Mechanical Properties of One-Part Alkali-Activated Copper Slag/Ground Granulated Blast Furnace Slag

**DOI:** 10.3390/ma14195505

**Published:** 2021-09-23

**Authors:** Patrick Ninla Lemougna, Nicole Dilissen, Guillermo Meza Hernandez, Felicite Kingne, Jun Gu, Hubert Rahier

**Affiliations:** 1 Department of Materials and Chemistry, Vrije Universiteit Brussel, Pleinlaan 2, 1050 Brussels, Belgium; Nicole.Dilissen@vub.be (N.D.); Guillermo.Meza.Hernandez@vub.be (G.M.H.); Felicite.Kingne.Kingne@vub.be (F.K.); Jun.Gu@vub.be (J.G.); Hubert.Rahier@vub.be (H.R.); 2 Strategic Initiative Materials in Flanders (SIM), 9052 Zwijnaarde, Belgium; 3 Department of Minerals Engineering, School of Chemical Engineering and Mineral Industries (EGCIM), University of Ngaoundere, Ngaoundere P.O. Box 454, Cameroon; 4 KU Leuven Department of Materials Engineering, Kasteelpark Arenberg 44, 3001 Leuven, Belgium

**Keywords:** copper slag, one-part geopolymer, microstructure, mechanical properties, building applications

## Abstract

Copper slag (CS) remains a challenging industrial by-product with a relatively small utilization fraction. The present study investigated the development of one-part alkali-activated cements based on CS, ground granulated blast furnace slag (GGBS) and a mixture of the two as a precursor. We investigated 5 to 15 wt% solid sodium metasilicate (Na_2_SiO_3_) and disilicate (Na_2_Si_2_O_5_) as alkaline reagents. Isothermal calorimetry showed that the reactivity of the system was higher for the metasilicate based samples, with early reaction and higher cumulative heat released. Metasilicate based samples also presented a more densified microstructure, lower porosity and higher strength. Better performances were observed with 10 wt% metasilicate/disilicate with respect to the 5 and 15 wt%. The 28-day compressive strength and elastic modulus of 10 wt% metasilicate samples reached 75 MPa and 25 GPa, respectively, and, for paste samples, ranged from 100 wt% GGBS to 50/50 wt% CS/GGBS. The microstructure and calorimetry of the pastes showed that GGBS actively participated in the binding process, whereas CS played a smaller role and acted as a filler and catalyst. The substitution of commercial GGBS by CS up to 50 wt% did not affect the overall performance, thus, bringing CS forward as an economically interesting precursor.

## 1. Introduction

Portland cement (PC) is a widely used material, with a global production estimated at 4.1 billion tons in 2017 [1]. However, with released CO_2_ concerns associated with PC production, there has been increasing interest in the development of alternative, more environmentally friendly binders, including geopolymers [2,3,4,5,6,7]. Alkali-activated materials (AAM) are defined as materials enclosing binder systems resulting from the reaction of a solid or dissolved alkali metal source, the activator, with a solid silicate powder [8]. Geopolymers are a subclass of alkali-activated materials. 

In addition to their interesting physical and mechanical properties, the possibility to use industrial residues in the development of geopolymers is advantageous for both a circular economy and the environment, preserving the limited natural resources used in building industry and tackling landfilling with sometimes hazardous waste [9,10,11,12,13,14]. However, one of the drawbacks associated with the development of AAM is the use of alkaline solutions in the synthesis process, i.e., one cannot simply add water to a powder. 

Hence, there has been increasing interest in the development of so called ‘one-part’ or ‘just add water’ geopolymers, mainly from industrial residues. Some of the materials investigated so far have included ground granulated blast furnace slag [15,16], mixtures of fly ash and slag [17,18,19], pre-treated wood biomass ash and diatomite [20], red mud [21], cement kiln dust and feldspar [22], lithium slag [23] and mining residues [24]. However, with the amount of 550–750 Mt/year of extractive waste generated in Europe [25] and the new European regulations encouraging the reuse of industrial side streams instead of landfilling [26], further research should aim to find industrial applications for these different side streams of extractive waste. 

Two such extractive wastes are copper slag (CS) and iron slag (GGBS). CS is generated during the production of copper at about 2.2 tons per ton of copper produced, with an annual generation estimated at about 24.6 million tons worldwide [27]. Despite several investigations on potential options for the use of CS, a large fraction is still landfilled [14,28,29,30]. Materialized real life applications of CS have only started recently to be explored [14,31], since the mineralogy and chemistry of CS, mainly made of CaO-FeOx-SiO_2_, may be less suitable in several cases as precursors for AAM. Therefore, more investigations should tackle the reaction mechanism, evolution of mechanical properties and performance of CS, in order to substantiate industrial applications and the commercialization of this particular extractive waste.

The use of GGBS, on the other hand, is well exploited up to commercialization and broad industrial applications [5,32]. The proven good reactivity of GGBS in alkaline systems may also be exploited for upcycling CS in alkali-activated blended systems based on CS/GGBS, as for the case of fly ash/GGBS, which has already led to commercial products [33,34]. Indeed, the compressive strength of GGBS based geopolymer at ambient temperature could easily reach 90 MPa [35]; in addition, while some alkali-activated synthetic iron rich slags were reported to reach 53 MPa at room temperature [14], industrial residues based on iron rich slag often need to be cured above ambient temperature to achieve a 28-day strength above 20 MPa [36].

Hence, the aim of this study was to investigate the development of one-part geopolymers from CS or blended CS/GGBS, and develop a mix design with a dry activator to just add water at ambient temperature. For reference purposes, some compositions made of only GGBS were prepared. Two solid sodium silicates with different modulus were investigated. The effect of a Blaine surface of the CS from 1500 to 4100 cm^2^/g as well as the effect of standard sand EN-196-1 addition to produce mortars were investigated on the compositions with highest compressive strength. 

The fresh geopolymer mixtures were characterized with several techniques, including isothermal calorimetry and setting time. The resulting cured geopolymer pastes and related mortars were characterized with optical microscopy, scanning electron microscopy, mercury intrusion porosity, compressive strength and Young’s modulus to assess their physical properties and potential suitability for building applications.

## 2. Materials and Methods

### 2.1. Materials

**Figure 1:**
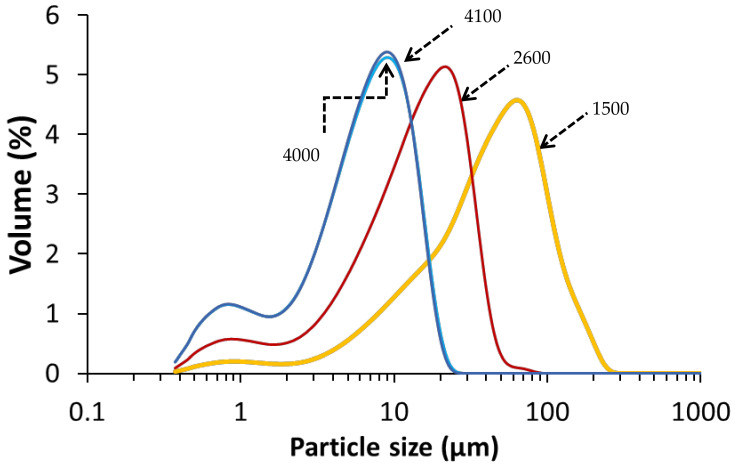
Particle size distribution of the CS per Blaine group, with the Blaine (cm^2^/g) indicated on the curves.

The copper slag (CS) used in this study was KORANEL® and was provided by Metallo, Beerse, Belgium. CS of 1500, 2600, 4000 and 4100 cm^2^/g Blaine surfaces were received in milled form from Metallo. The Blaine was measured with an air permeability apparatus as described in EN 196-6:2018. The particle size distribution of each CS Blaine group (Figure 1) was determined by a laser particle size analyzer (Beckman Coulter LS 13320, Brea, CA, USA) at the department of Materials Engineering (MTM, KU Leuven, Belgium). The chemical composition of CS as determined by X-ray fluorescence (PW 2400 Philips; MTM, KU Leuven) on powder is presented in Table 1. 

**Figure 2:**
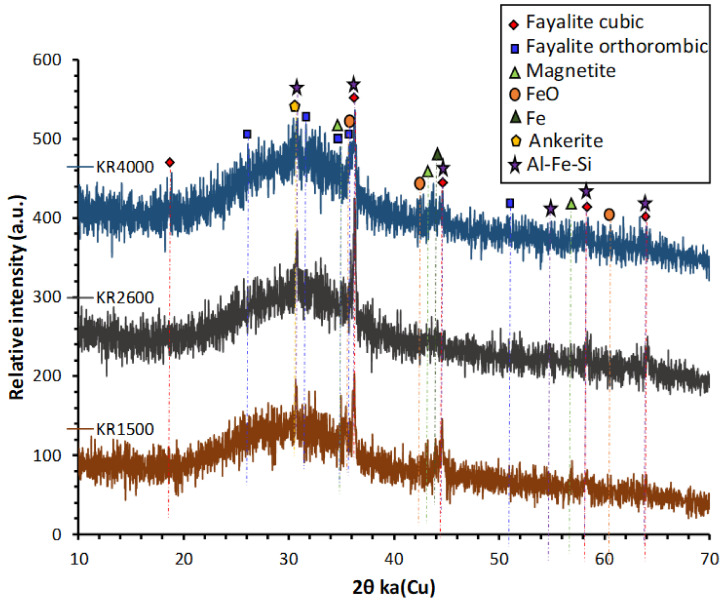
XRD diffractograms of KORANEL® (KR) samples of 1500, 2600 and 4000 Blaine.

The X-ray powder diffraction (XRD) spectra of the CS were taken by D2 Phaser (Bruker; MTM, KU Leuven, Belgium) in the 6–70° 2θ range using Cu Kα radiation (voltage 40 kV and current 40 mA), a step size of 0.02° and scan speed of 0.5 s/step. XRD shows that it was mainly amorphous, with few crystalline features mainly ascribed to fayalite and magnetite (Figure 2). The GGBS used in this study was ECOCEM from the ORCEM company, Moerdijk 4782 SK, Netherlands. The chemical composition of GGBS is given in Table 1. Sodium metasilicate, Na_2_SiO_3_ (47 wt% SiO_2_, 50 wt% Na_2_O, 97 wt% dry matter) and sodium disilicate, Na_2_O.2SiO_2_ (54.5 wt% SiO_2_, 27.5 wt% Na_2_O, 82 wt% dry matter) were supplied by Silmaco, NV, Industrieweg 90, 3620 Lanaken, Belgium.

### 2.2. Samples Preparation

CS with a Blaine of 2600 cm^2^/g, GGBS and mixtures of the two with alkaline reagents were dry mixed. The details on the mix design are summarized in Table 2. Demineralized water was added to the dry mixture with a water to powder ratio (W/P) of 0.40 and 0.32 for the GGBS and CS precursors, respectively, and 0.33 for the admixtures to obtain a homogeneous slurry during mixing. The different liquid to solid ratios were adopted after preliminary experiments aiming to get compositions with approximately the same workability.

Additionally, the effect of the copper slag Blaine specific surface area (1500, 2600, 4000 and 4100 cm^2^/g) and CEN standard sand (EN 196-1) addition for the production of mortar were investigated on selected disilicate and metasilicate compositions 8: DS10-CS50 and MS10-CS50, respectively. For the preparation of the mortar, the water to powder ratio was adjusted to 0.46 for all the samples, while the pastes maintained the same ratio as in Table 2. The fresh mixtures were mixed at 20 °C according to the methodology EN 196-1, casted in cubic molds (3.5 × 3.5 × 3.5 cm^3^), sealed in PET-foil and cured for 28 days at 20 °C.

### 2.3. Characterization Methods

Isothermal (20 °C) calorimetry (TAM Air, TA instruments, 159 Lukens Drive, New Castle, DE, USA) was performed on 10 g of the solid part plus added water according to the W/P ratio in Table 2. It was mixed outside the calorimeter in the ampoule and placed within the calorimeter after 2 min of mixing at 1600 rpm. The data collection started 45 min after the start of mixing.

For microstructural and chemical elemental analyses, mm-sized sample chunks were embedded in epoxy. To reach the surface, samples were shortly grinded by hand with Grit 1200 and 4000 Silicon Carbide grinding paper and water. Then, they were polished with 3 µm diamond in water suspension for 5 min at 20 kN and 150 rpm disc speed and in the same direction 150 rpm head speed, followed by 1 µm diamond in water suspension for 5 min at same conditions. Optical microscopy was performed on these polished samples with an Axiocam ERc 5s (ZEISS, Carl-Zeiss-Strasse 22, Oberkochen, Germany) in gray scale colors. 

After overnight vacuum, they were prepared for scanning electron microscopy (SEM) and coated with Au. The analyses occurred with combined energy-dispersive X-ray spectroscopy (EDS) and SEM using a FEI XL30 FEG microscope (Thermo Fischer Scientific, Waltham, MA, USA). The conditions were at 10^−5^ mbar pressure, an acceleration voltage of 10 kV and probe current spot size of 3.0 for secondary electron (SE) and backscatter electron (BSE) imaging.

Mercury intrusion porosimetry (MIP) was performed to gain insight into the porosity size distribution of the samples. Before starting those analyses, a vacuum saturation test was performed to gain insight on the bulk density and open porosity of the samples. This was used to identify the amount of sample that could be used during the MIP measurements. For the vacuum saturation test, the dry mass (*m_dry_*) of the samples was obtained after heating the samples for 9 h at 60 °C. Then, the samples were put in a desiccator, and a vacuum was pumped for 3 h. After that, demineralized water entered the desiccator at a flow rate of 5 cm/h to merge the samples until 5 cm underneath the water level. After the samples were kept under water for 24 h, their wet mass (*m_wet_*) was weighed immediately, as well as the underwater mass (*m_under_*). The bulk volume (*V_bulk_*; m^3^), bulk density (*ρ_bulk_*; kg/m^3^) and open porosity (φ;%) are calculated as:

(1) $$V_{bulk}=\frac{m_{wet}+m_{under}}{\rho_{water}}$$

(2) $$\rho_{bulk}=\frac{m_{dry}}{V_{bulk}}$$

(3) $$\varphi =\frac{m_{wet}-m_{dry}}{m_{wet}-m_{under}}\times 100$$

The open porosity and bulk density were used to calculate the mass of sample to use for MIP, in order that the stem volume used at the end of the measurement was >25% and <90% to have a reliable measurement. Masses between 2.15–2.85 g were used in a penetrometer type 7 with stem volume of 0.392 mL. MIP was conducted using Auto Pore Ⅳ 9500 (Micrometrics, 4356 Communications Drive, Norcross, GA, USA). Low pressure measurement occurred 0 to 30 psia for pore diameter range of 360–3.6 µm followed by a high pressure measurement at 15 to 30,000 psia for a pore diameter range of 6 to 0.0055 µm.

Compressive strength and elastic modulus were performed as described in Methods of testing cement standard (EN196-1: 2016). The elastic modulus test was performed prior to the compression test since this is a non-destructive technique. An ultrasonic pulse analyzer (Pulsonic, Controls, Via Salvo D’Acquisto, 2 Liscate, Milan, Italy) was used to measure the transit time in the samples. Knowing the dimensions of the specimens, it was possible to estimate the pulse velocity. With the equation presented below, the E modulus is calculated. C: pulse velocity; *ρ*: bulk density.

(4) $$c=\sqrt{\frac{E}{\rho }}$$

The compressive test was performed with an Instron 5885H with a maximum load of 250 kN, and the displacement rate of the head was 1 mm/min until failure of the sample. The compressive strength ($\sigma_{max}$) was calculated by acquiring the maximum load ($F_{max}$) applied on the sample and dividing by the cross sectional area (A) of the specimen:

(5) $$\sigma_{max}=\frac{F_{max}}{A}$$

Three (03) specimens were measured for each composition and curing time. The error bar is the standard deviation of replicate specimens.

## 3. Results and Discussion

### 3.1. Mechanical Properties

**Figure 3:**
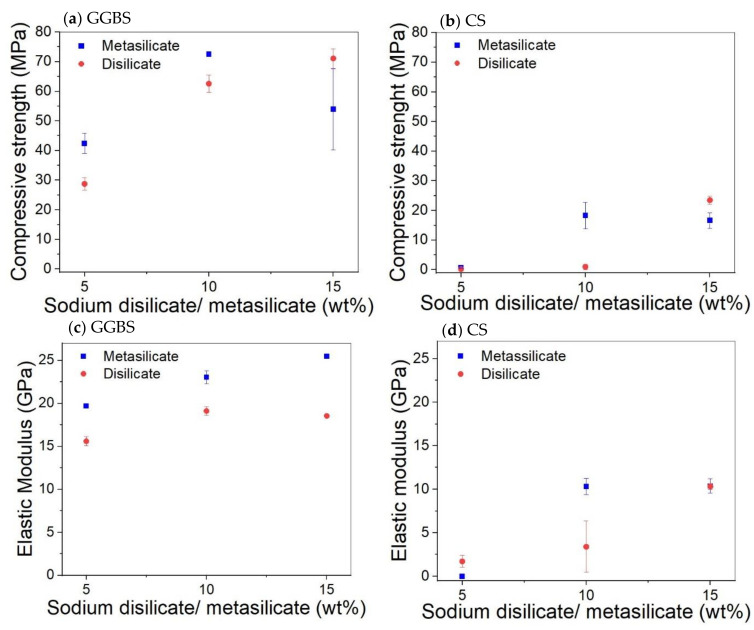
Effect of the amount of sodium disilicate/metasilicate on the compressive strength and elastic modulus of GGBS (**a**,**c**) and CS (**b**,**d**) based systems at 28 days.

The mechanical properties of compositions involving only GGBS or CS (1: DS/MS5-CS0 to 6: DS/MS15-CS100; Table 2) are presented in Figure 3. The compressive strength of pure GGBS samples increases almost linearly with the amount of activator until 10 wt% and then decreases again. As the disilicate has a lower amount of Na, it was expected to be less activating, which is evident at 5 and 10 wt% of activator. On top of that, the disilicate has a slightly higher water content as shown by the liquid to solid ratio (L/S) with respect to metasilicate (L/S DS vs. MS; Table 2); however, this cannot explain a difference of more than 10% in strength. 

For the highest amount of activator (15 wt%), the strength of the disilicate sample equals the one of the 10 wt% metasilicate, although the amount of Na in the system is still not the same. This difference between metasilicate and disilicate is also observed for CS, and thus to obtain the same strength, more disilicate needs to be added. The compressive strength for the CS samples is much lower. To build up significant strength in 28 days, 5 wt% of silicate is not sufficient. The disilicate gives again lower strength for 10 wt%, but gives a higher strength at 15 wt%. The elastic modulus results of samples made of GGBS and CS kept almost the same trend as for the compressive strength results.

**Figure 4:**
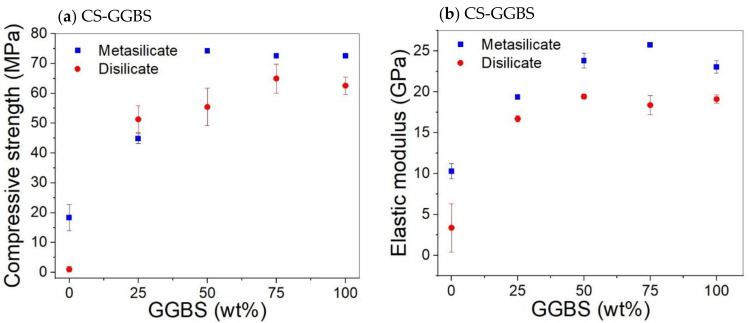
Effect CS substitution on the compressive strength (**a**) and elastic modulus (**b**) of CS/GGBS based system with 10 wt%. DS/MS.

The compressive strength and elastic moduli of CS/GGBS mixtures are presented in Figure 3. The substitution of 25 wt% CS by GGBS (compositions 7: DS10-CS75 and MS10-CS75; Table 2) led to a significant increase in both compressive strength and elastic modulus, in comparison to the system based only on CS (Figure 4). The compressive strength increased from about 0 to 50 MPa for the sample prepared with 10 wt% sodium disilicate (DS10-CS75) and 20 to 45 MPa for samples prepared with sodium metasilicate (MS10-CS75) (Figure 4).

Figure 4 shows that substitution of CS by GGBS led to an increase of the compressive strength, with a general trend of higher strength for samples prepared with MS. This is ascribed to a higher gel formation due to higher sodium content in MS based samples as shown by the Na/Al molar ratios in Table 2. However, the extent of the increase is not up to the approximately two times difference when compared to the amount of Na in MS/DS based samples. 

Hence, DS based samples could be considered as more economic and environment friendly. The Na/Al molar ratios were in the range 0.57–0.61 in DS based systems containing CS/GGBS and 1.03–1.11 in MS based systems containing CS/GGBS. For a simpler precursor, like metakaolin, the Na/Al molar ratio of about 1 was observed to be optimum for the mechanical properties [37]. However, this could not be applied in CS/GGBS systems where the reaction products are influenced by reactive calcium and iron [4,13].

**Figure 5:**
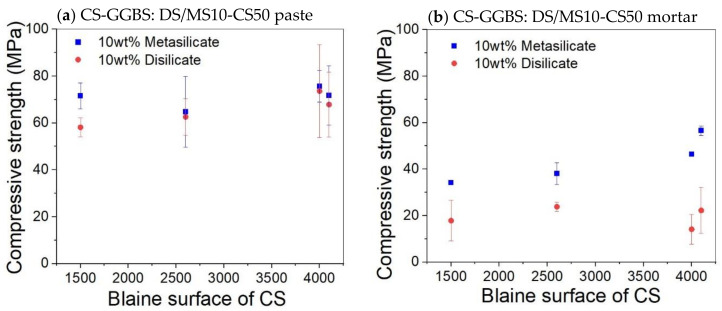
Effect of Blaine surface on the compressive strength of paste (**a**) and mortar (**b**) based on composition 8: DS10-CS50 and MS10-CS50.

No significant change on the compressive strength was observed on varying the Blaine surface for both pastes and mortar (Figure 5). It was expected that a higher specific surface area would lead to faster strength build up and even a higher final strength. The fact that this is not the case means that the extra grinding is not beneficial. A coarser slag requires less grinding energy and, thus, a lower economic and ecologic cost. The workability will also be better. 

The addition of sand for the preparation of mortar led globally to samples with reduced mechanical properties. The compressive strength of the disilicate and metasilicate mixture compositions DS10-CS50 and MS10-CS50 were above 50 MPa, while those of mortars based on metasilicate were above 30 MPa (Figure 5), satisfying the requirements for building materials according to ASTM C62. Mortar mixtures based on disilicate still reached a compressive strength around 20 MPa, still fulfilling applications, such as floors or non-supporting walls.

### 3.2. Calorimetry

**Figure 6:**
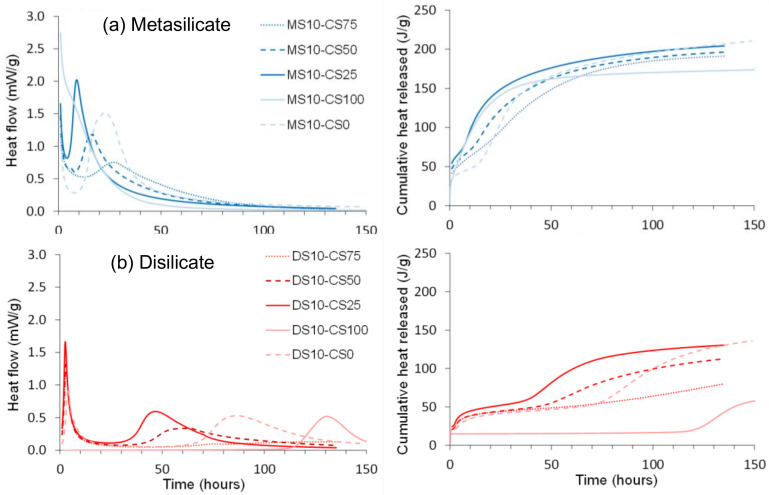
Normalized heat flow and cumulative heat released of (**a**) disilicate (DS) and (**b**) metasilicate (MS) based systems.

A calorimetry study was performed on pure CS and GGBS systems as well as CS/GGBS based systems (Figure 6).

The normalized and cumulative heat flow showed a higher heat released with the MS based samples. This is ascribed to a higher content in sodium that likely led to higher alkalinity and dissolution of the geopolymer precursors after mixing with water and, thus, a higher reactivity.

GGBS reacted in two sequences clearly observable for the pure GGBS-DS system, as well as in the blended DS systems (Figure 6a). The first peak around 1 h after mixing may be ascribed to dissolution starting at the surface of the particles. This is succeeded by a dormant period, comparable to what is observed for OPC. The second exotherm at 80 h for pure GGBS-DS system and at 40 to 70 h for blended DS systems after mixing releases more heat probably linked to the reaction of a larger fraction of the material. The surface reaction of the MS based samples (Figure 6b) started prior to the start of the calorimetry measurement and, therefore, could not be recorded completely, whereas the second exotherm, linked to the reaction of a larger fraction of the material was recorded at 10 to 30 h.

At variance to the pure GGBS-DS system, which presented two peaks, pure CS and DS only presented one peak around 130 h (Figure 6a). As for the case of pure CS and GGBS systems, a higher heat release was observed with MS-based samples for the mixture of CS/GGBS systems. The trend for two sequential reactions as for GGBS was maintained. A higher heat release was observed with the increase of GGBS in the system, confirming the higher reactivity of GGBS.

Adding CS to GGBS, as well in DS as in MS, shifts the first and second exotherm to shorter times. This indicates that CS is acting as a catalyst for the reactions. Only for 75 wt% of CS, the second exotherm comes after the second exotherm for the pure GGBS. This could be due to the use of most of the silicate solution for the GGBS reaction, resulting in a lower concentration for the second part of the reaction. It is clear that the CS has an important influence on the GGBS reaction, but the exact reactions going on need further investigation. It is namely consistent in the blends that the higher the GGBS content, the earlier the peak whereas pure GGBS is reacting slower). 

Hence, a small amount (25 wt%) of CS in the GGBS system catalyzes the reaction and sharpens the reaction peak, but the more CS (50 to 75 wt%) is added, the more it delays and broadens the peak of the catalyzed reaction. The cumulative heat flow is higher for GGBS dominant systems and decreases with higher amounts of CS. The higher heat released in samples containing a higher amount of GGBS is consistent with the compressive strength results presented in Figure 3 and Figure 4 where higher strength values were observed for systems containing GGBS.

### 3.3. Porosity and Bulk Density

For porosity measurements, the samples with best performance (composition 8: DS10-CS50 and MS10-CS50 and composition 9: DS10-CS25 and MS10-CS25 for mixtures of CS and GGBS and composition 2: DS10-CS0 and MS10-CS0 and composition 3: DS15-CS0 and MS15-CS0 for GGBS as reference) were selected (see Table 2 for nomenclature). No DS/MS system with pure CS was selected due to low performance.

**Figure 7:**
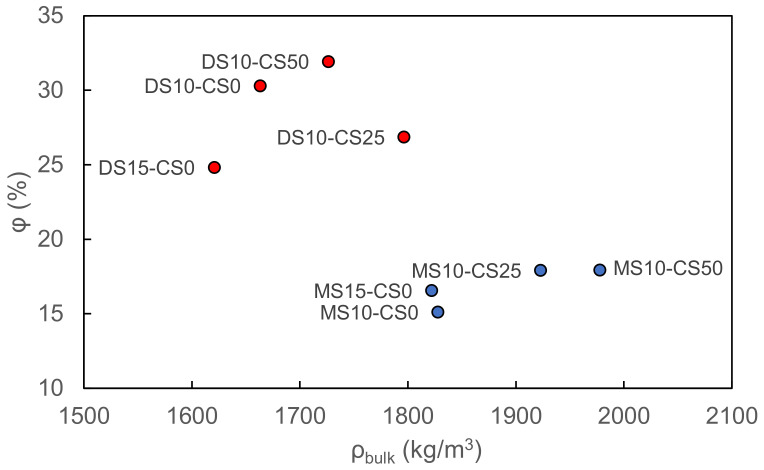
The porosity vs. bulk density of metasilicate and disilicate samples (2) DS10-CS0 and MS10-CS0, (3) DS15-CS0 and MS15-CS0, (8) DS10-CS50 and MS10-CS50 and (9) DS10-CS25 and MS10-CS25 (see Table 2 for mix design) based on vacuum saturation.

The vacuum saturation test reveals a higher porosity and lower bulk density for disilicate samples with respect to metasilicate samples. Disilicate samples namely vary between 25–32 pore vol% and 1.6–1.8 g/cm^3^ bulk density whereas metasilicate samples vary between 15–18 pore vol% and 1.8–2.0 g/cm^3^ bulk density. The difference in density and porosity can be related to a higher dissolution and matrix formation in metasilicate based samples. The addition of CS to the GGBS system shows both for meta- and disilicate samples an increase in bulk density, but not necessarily a change in porosity, based on the vacuum saturation test (Figure 7). The increased bulk density can be related to the Fe-content in the CS.

**Figure 8:**
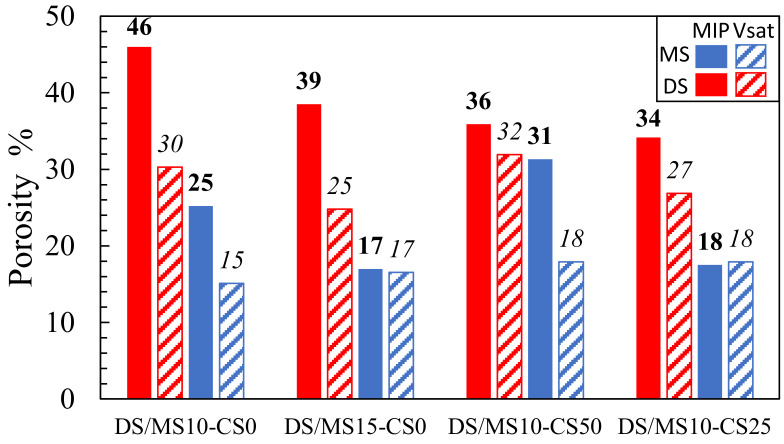
Porosity based on mercury intrusion porosimetry (MIP; values in bold) and vacuum saturation test (Vsat; values in italic) for samples (2) DS10-CS0 and MS10-CS0, (3) DS15-CS0 and MS15-CS0, (8) DS10-CS50 and MS10-CS50 and (9) DS10-CS25 and MS10-CS25 (see Table 2 for mix design).

Both the vacuum saturation and MIP showed consistently that disilicates had a higher porosity than metasilicate samples (Figure 8). The MIP further showed that an increase in DS/MS resulted in a lower porosity, which is not directly obvious in the vacuum saturation (composition 2: DS10-CS0 and MS10-CS0 vs. composition 3: DS15-CS0 and MS15-CS0; (Figure 8). The reduction in porosity with the increase in DS/MS is ascribed to a higher extend of reaction. The mixtures of CS and GGBS (composition 8: DS10-CS50 and MS10-CS50 and composition 9: DS10-CS25 and MS10-CS25; Figure 8) might result in a lower porosity with respect to pure GGBS (composition 2: DS10-CS0 and MS10-CS0; Figure 8), which is not visible with the vacuum saturation test. Since 46% porosity for DS10-CS0 is rather high and there is no full agreement between MIP and the vacuum saturation test and the density, we may say that the MIP measurements may be difficult to interpret.

The higher values for MIP with respect to the vacuum saturation test might be due to the smaller pore sizes, which can be filled with mercury intrusion under high pressure and not with water intrusion under ambient pressure. This shows that smaller pores remain within pure GGBS DS/MS mixtures, whereas, when adding the CS, these pores can be filled. Adding CS to the GGBS system may, therefore, have influence on strength development if they had comparable reactivity.

**Figure 9:**
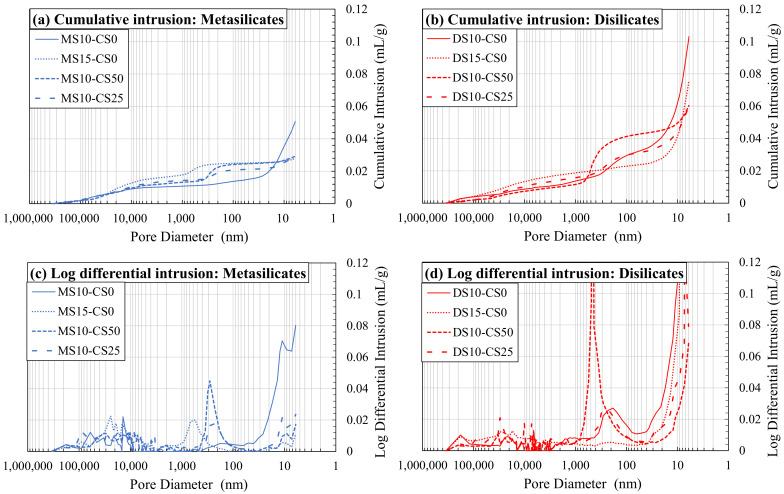
Mercury intrusion porosimetry (MIP) as cumulative intrusion (**a**,**b**) and log differential intrusion (**c**,**d**) in function of pore diameter for metasilicates (**a**,**c**) and disilicates (**b**,**d**) (See Table 2 for mix design).

This can also be seen in Figure 9. A higher amount of DS/MS shows coarser pores and less fine pores (composition 2: DS10-CS0 and MS10-CS0 vs. composition 3: DS15-CS0 and MS15-CS0; Figure 9a,b), although, again, the MIP results were likely affected by the bottle neck effect. The addition of CS did not appear to influence the coarser pore distribution, but moderately increased the amount of pores between 1000–100 nm and significantly lowered the amount of pores smaller than 20 nm. If a higher amount of CS was added, the amount of pores between 1000–100 nm increased even more (composition 8: DS10-CS50 and MS10-CS50 vs. composition 9: DS10-CS25 and MS10-CS25 Figure 9c,d). 

It could be concluded that the addition of CS to GGBS in meta- and disilicates lowers the fine porosity, e.g., for 25 wt% substitution, and creates a porosity around 1000–100 nm when a higher amount is added, e.g., for 50 wt% substitution. This filler effect of CS can, thus, be interesting since small pores also influence the mechanical properties. When compared to the compressive strength of the mixtures with 25 to 50 wt% CS (55–75 MPa; Figure 4) to 0% addition CS (60–70 MPa; Figure 3), we see favorable behavior, i.e., the strength remains constant. This means that a cheaper material can be created with same performance when substituting GGBS by up to 50 wt% of CS, due to the filler effect, in combination with a limited reactivity of CS.

### 3.4. Microstructural Characterization

**Figure 10:**
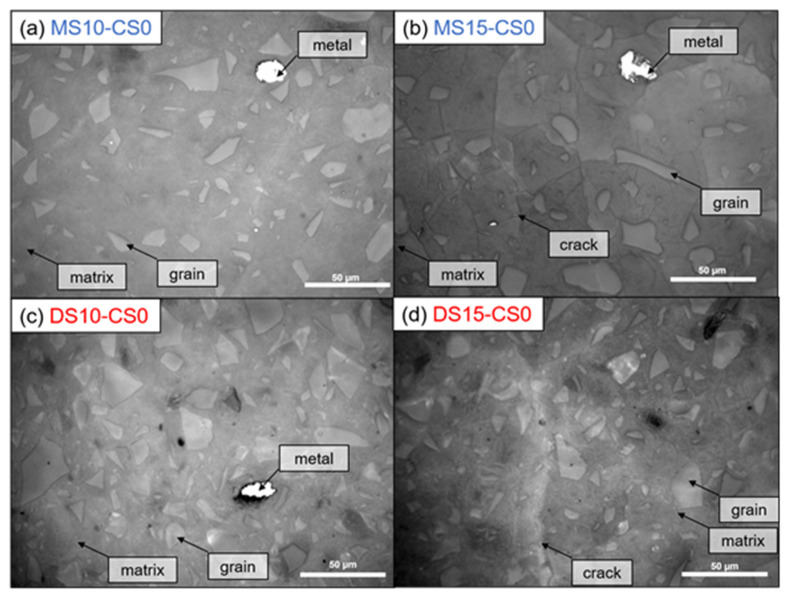
Optical microscopy with incident light. White is Fe-metal, light gray are GGBS grains, darker gray is the matrix, and dark lines are cracks (See Table 2 for mix design).

Optical microscopy shows no cracks within the 10 wt% DS/MS mixtures, whereas cracks were visible within the 15 wt% DS/MS mixtures (Figure 10). These cracks are likely to be created due to the coarser pore system for 15 wt% DS/MS mixtures that could have been more susceptible to cracking than the finer pore system for the 10 wt% DS/MS mixtures. A note with the imaging is that DS10-CS0 shows a solid, non-porous structure, which does not perfectly agree with the MIP result (46 pore vol%; (Figure 8). Seeing that the addition of CS would only fill up the finer pores, it likely does not prevent the cracking as the amount of coarser pores remains unchanged. 

Nevertheless, 10 wt% DS/MS has similar strength development than 15 wt%, meaning that if cracking could be decreased within the 15 wt% DS/MS systems, they might develop a higher strength. The fact that 15 wt% DS/MS systems show lower porosity than the 10 wt% DS/MS systems (composition 2: DS10-CS0 and MS10-CS0 vs. composition 3: DS15-CS0 and MS15-CS0; (Figure 8), also supports that strength might be higher for the 15 wt% if cracks would be absent. In the current research, the 10 wt% DS/MS systems are more of interest due to the lower susceptibility to cracking and the lower cost.

**Figure 11:**
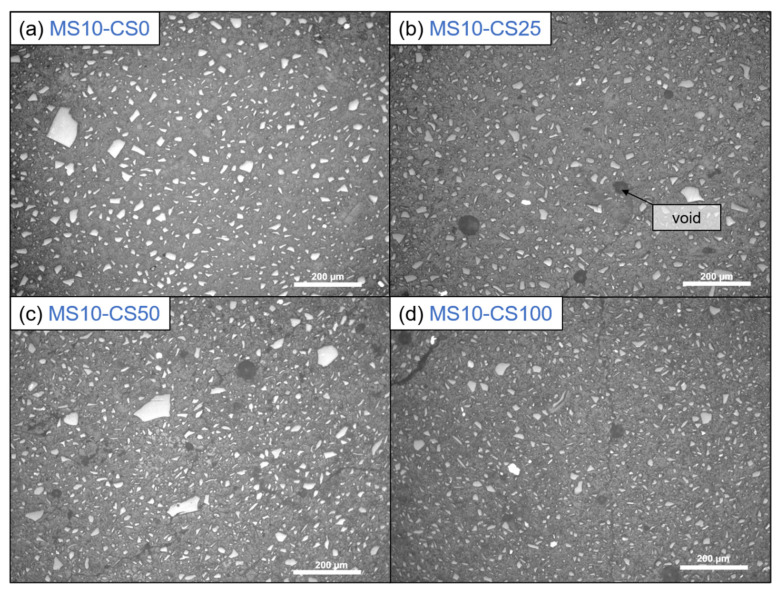
Optical microscopy with incident light of 10 wt% metasilicate systems. Slag composition varies from (**a**) composition 2: MS10-CS0, 100 wt% GGBS, (**b**) composition 9: MS10-CS25, 75 wt% GGBS, 25 wt% CS, (**c**) composition 8: MS10-CS50, 50 wt% GGBS, 50 wt% CS to (**d**) composition 5: MS10-CS100, 100 wt% CS. Dark gray bubbles are voids. (See Table 2 for mix design).

Optical microscopy shows that the presence of CS creates voids in the systems (Figure 11). When 100 wt% GGBS as slag is used with 10 wt% of disilicate, namely, no microscopic voids are observed, whereas when 25 wt% to 100 wt% of GGBS is substituted by CS, up to 50 µm-sized voids are observed. The addition of CS, thus, either causes gas formation that is trapped during setting, slightly fosters air entrainment, or a combination of both. A formation of gas might also explain the higher amount of pores around 1000–100 nm with higher amount of CS (composition 8: DS10-CS50 and MS10-CS50 vs. composition 9: DS10-CS25 and MS10-CS25 (Figure 9c,d). This gas is likely formed upon oxidation of metallic inclusions in the slag, or the oxidation of Fe^2+^, as it is originally present in CS, to Fe^3+^. All disilicate systems, on the other hand, have voids. It is also clear from Figure 8 that disilicates have higher porosity than metasilicates. This is in line with the development of compressive strength, which is higher for metasilicates (Figure 3 and Figure 4).

**Figure 12:**
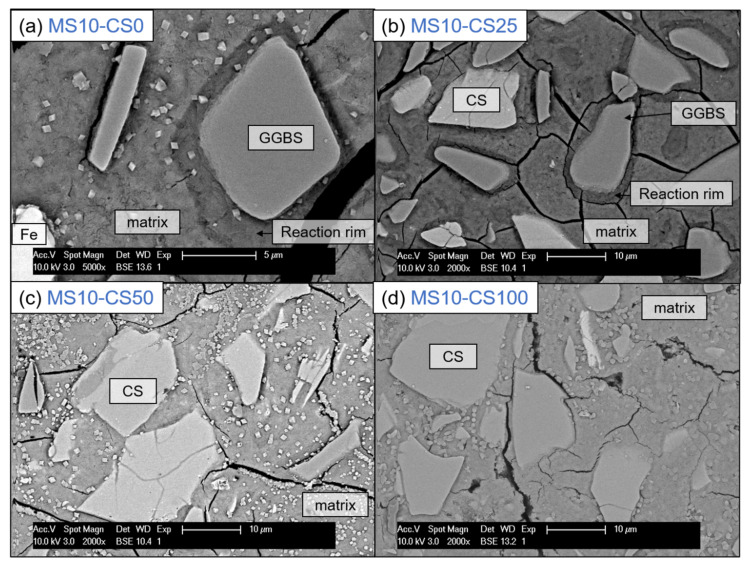
SEM-BSE imaging at 5000× (**a**) and 2000× (**b**–**d**) magnification of 10 wt% metasilicate systems. Slag composition varies from (**a**) 100 wt% GGBS, 2-MS10CS0, (**b**) 75 wt% GGBS, 25 wt% CS, 9-MS10CS25, (**c**) 50 wt% GGBS, 50 wt% CS, 8-MS10CS50 to (**d**) 100 wt% CS, MS10CS100. Cracking observed can be due to vacuum treating the samples before BSE analyses. Magnification of (**a**) is higher to show the reaction rim enlarged (See Table 2 for mix design).

BSE imaging of the GGBS disilicate systems shows a reaction rim around the grains, indicating clearly that the GGBS actively dissolves (Figure 12a). This is also evident for slag fines that have been completely reacted and leave behind a darker gray-scale patch in the matrix [38]. When replacing 25 wt% of the slag by CS, this reaction rim and patches are present for GGBS (Figure 12a). The CS grains on the other hand do not show a rim, and this is irrespective of the percentage of replacement (Figure 12b–d). This difference between GGBS and CS clearly shows the difference in reaction mechanism between the two slags, but can also be due to the reactivity for which the GGBS more actively contributes in dissolving and bonding with the matrix. 

This latter is also obvious when observing the cracking behavior: when a crack is reaching the reaction rim around the GGBS grain, it does not just continue until the grain, but clearly continues around the rim (see crack around GGBS grain in the middle of (Figure 12b), whereas a CS grain will be separated from the matrix by a propagating crack (see crack around the CS grain in the middle of Figure 12d). This shows one reason why a pure CS system is much weaker in strength than when involving GGBS to the system (*cf* Figure 3 and Figure 4).

**Figure 13:**
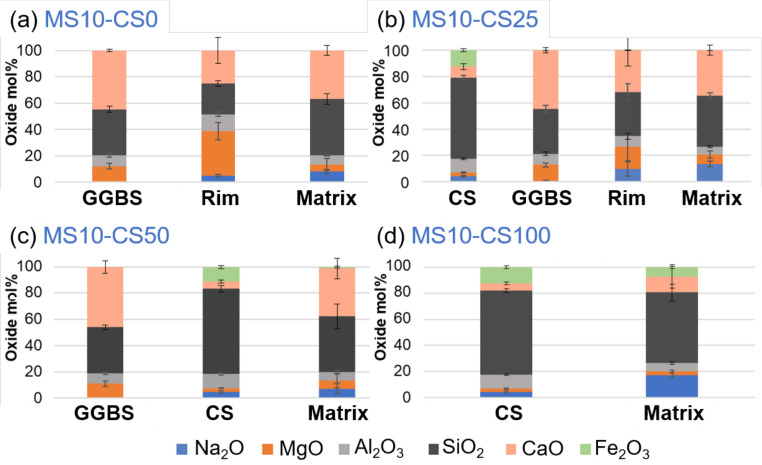
EDS-SEM of 10 wt% metasilicate systems. Slag composition varies from (**a**) 10 wt% GGBS, 75 wt% GGBS, (**b**) 25 wt% CS, 50 wt% GGBS, (**c**) 50 wt% CS to (**d**) 100 wt% CS (see Table 2 for mix design).

EDS-SEM analyses of the metasilicate GGBS systems show a higher Mg- and Al-content and lower Si- and Ca-content within the rim (Figure 13; *cf* rim in Figure 12a,b). This is typically observed within slag cements that are activated with Na, with hydrotalcite and AFm phases in the rims ([38,39] and the references therein). With respect to the matrix, it has a lower Na-content. This surrounding of Mg-Al-network shows the reactivity of the GGBS grains with the metasilicate. As the metasilicate fully dissolves in water, it activates the dissolution of the GGBS grains. 

Mg and Al will be less mobile and concentrate within a rim around the grains to react with Na of the disilicate solution, whereas Ca seems more mobile to react with the metasilicate solution in a matrix further from the GGBS grains (Figure 13a). If 25 wt% of GGBS is replaced by Fe-rich CS (Figure 13b), similar behavior is observed for the GGBS rims and matrix. The Fe present within the CS grain does not seem to appear within the matrix. However, when the CS replacement is increased to 50 wt%, a fraction of Fe is detected within the matrix (Figure 13c). CS thus also reacts with the metasilicate solution and Fe can actively participate within the matrix-bonding. 

This becomes even more obvious when 100 wt% CS is used within the metasilicate system (Figure 13d) as the matrix shows an even higher content of Fe. This agrees with iron behavior in alkali activation of some iron rich slags [4,40]. The higher Ca-content within the matrix with respect to the grain of the CS-metasilicate system likely supports the bonding within the matrix. The reactivity of CS and bonding between CS and matrix is weak (*cf* Figure 13d).

If both slags are introduced into the metasilicate system, the preferential dissolution of GGBS grains will occur, while CS grains react much slower, and its contribution to the matrix is only detected for higher CS content (i.e., minimal Fe is detected for 50 wt% replacement but not for 25 wt% replacement; Figure 13b,c). This was not expected in view of the calorimetry data showing a catalyzing effect of CS on GGBS. A higher Ca-content in the matrix, thus, appears to be essential for the stronger bonding and strength development within the matrix (see Ca-content in 100 wt% CS systems compared to systems containing GGBS Figure 13). CS, thus, adds to the system mainly as fine pore filler with minor bonding on nanoscale when 50 wt% or higher of the slag is CS.

In the disilicate systems, the same as within the metasilicate system can be observed, microstructurally as well as chemically. Rims around GGBS grains show higher Mg- and Al-content and lower Si- and Ca-content. Fe is not detected within the matrix except for GGBS replacement by 50 wt% CS and higher. The Ca-content within the matrix is higher when GGBS is involved within the system with respect to 100 wt% CS in the disilicate system. Therefore, same conclusions may be drawn. If directly comparing metasilicate systems to disilicate systems then a higher Ca-content is observed within the matrix of the metasilicate systems. This might be ascribed to a higher dissolution of the starting precursors within metasilicate systems, which also lowered the porosity (Figure 8).

## 4. Conclusions

The present study investigated the development of one-part geopolymers based on CS, GGBS, sodium metasilicate (Na_2_SiO_3_) and disilicate (Na_2_Si_2_O_5_). The reactivity of the systems was investigated by isothermal calorimetry, and the products were characterized by optical microscopy, scanning electron microscopy, mercury intrusion porosity and setting time. The 28-day compressive strength reached was as high as 75 MPa, and the elastic modulus was as high as 25 GPa with the composition made of 50/50 wt% CS/GGBS. Replacement of 25 wt% of CS by GGBS resulted in more than doubling the strength and E-modulus compared to the pure CS system. Higher density, lower porosity and better performances were observed with sodium metasilicate at an optimum value around 10 wt%. 

The use of standard sand to produce mortar-based samples led to compressive strengths of around 40 MPa. No significant differences in strength were observed when changing the specific surface area of CS. BSE and EDS-SEM showed a reaction rim around the GGBS grains and not around the CS grains. The chemical composition in the matrix showed a larger participation of GGBS compared with CS, and, for higher amounts of CS (>50 wt%), Fe was observed in the matrix. This brings CS forward as a filler more than as an active binder. When comparing the compressive strength of the mixtures with 25 to 50 wt% CS (55–75 MPa) to 0% addition CS (60–70 MPa), we saw favorable behavior, i.e., the strength remained constant. 

This means that a cheaper material can be created with same performance when substituting GGBS by up to 50 wt% of CS due to the filler effect without reducing the mechanical performance. Calorimetry confirmed the faster reaction of the pure GGBS system in comparison to the pure CS system. However, CS acted as catalyst in the system based on CS/GGBS, shortening the reaction time in comparison to the pure GGBS system. The results obtained are of interest for the management of CS and its upcycling in cementitious materials for building applications. Further investigation on the durability of the materials in various environments will be of interest.

## Figures and Tables

**Table 1:** Normalized chemical composition in wt% by XRF analyses for CS. * includes small amounts that are <0.1 wt%. GGBS is according to the technical datasheet of Ecocem (www.ecocem.ie, accessed on 1 June 2021).

Samples	SiO_2_	Al_2_O_3_	Fe_2_O_3_	CaO	MgO	Cr_2_O_3_	MnO	K_2_O	TiO_2_	P_2_O_5_	SO_3_	Others *
CS	24.8	8.9	53.6	2.6	0.9	0.7	-	0.2	0.2	0.7	0.8	0.06
GGBS	36.5	10.4	0.7	42.4	8.1	-	0.4	-	0.5	-	0.7	-

**Table 2:** Mix design of the prepared one-part geopolymers. DS and MS stand for sodium disilicate and sodium metasilicate, respectively. CS 2600 is the copper slag with a Blaine of 2600 cm^2^/g. W/P is the water to powder ratio based on the amount of added water; and the real liquid/solid (L/S) is calculated based on added water and water present in the DS/MS.

Composition Code	Solid Na-DS/MS (g)	GGBS (g)	CS 2600 (g)	Na/Al (Molar)		W/P	L/S	
				DS	MS		DS	MS
(1) DS/MS5-CS0	5	95	0	0.26	0.47	0.40	0.41	0.40
(2) DS/MS10-CS0	10	90	0	0.54	0.99		0.43	0.40
(3) DS/MS15-CS0	15	85	0	0.84	1.47		0.44	0.41
(4) DS/MS5-CS100	5	0	95	0.30	0.55	0.32	0.33	0.32
(5) DS/MS10-CS100	10	0	90	0.64	1.16		0.34	0.32
(6) DS/MS15-CS100	15	0	85	1.18	1.84		0.36	0.33
(7) DS/MS10-CS75	10	22.5	67.5	0.61	1.11	0.33	0.34	0.33
(8) DS/MS10-CS50	10	45	45	0.59	1.07		0.35	0.33
(9) DS/MS10-CS25	10	67.5	22.5	0.57	1.03		0.37	0.34

## Data Availability

The data presented in this study are available on request from the corresponding author.
